# Of Screening, Stratification, and Scores

**DOI:** 10.3390/jpm11080736

**Published:** 2021-07-28

**Authors:** Bartha M. Knoppers, Alexander Bernier, Palmira Granados Moreno, Nora Pashayan

**Affiliations:** 1 Centre of Genomics and Policy, Faculty of Medicine, McGill University, 740 Avenue Dr. Penfield, Suite 5200, Montreal, QC H3A 0G1, Canada; alexander.bernier@mcgill.ca (A.B.); palmira.granadosmoreno@mcgill.ca (P.G.M.); 2 Department of Applied Health Research, University College London, 1-19 Torrington Place, London WC1E 7HB, UK; n.pashayan@ucl.ac.uk

**Keywords:** bioethics, cancer screening, law, stratification, personalized medicine, polygenic risk scores

## Abstract

Technological innovations including risk-stratification algorithms and large databases of longitudinal population health data and genetic data are allowing us to develop a deeper understanding how individual behaviors, characteristics, and genetics are related to health risk. The clinical implementation of risk-stratified screening programmes that utilise risk scores to allocate patients into tiers of health risk is foreseeable in the future. Legal and ethical challenges associated with risk-stratified cancer care must, however, be addressed. Obtaining access to the rich health data that are required to perform risk-stratification, ensuring equitable access to risk-stratified care, ensuring that algorithms that perform risk-scoring are representative of human genetic diversity, and determining the appropriate follow-up to be provided to stratification participants to alert them to changes in their risk score are among the principal ethical and legal challenges. Accounting for the great burden that regulatory requirements could impose on access to risk-scoring technologies is another critical consideration.

## 1. Introduction

“All Screening Programmes Do Harm; Some Do Good as Well, and of These, Some Do More Good than Harm at Reasonable Cost” [1].

By 2025, 130 million genomes are expected to be sequenced, of which 83 million will be cancer genomes [2]. Whole genome sequencing (WGS) results, when combined with other real-world clinical and socio-demographic data, will allow for ongoing risk stratification and re-classification with prognostic value not only for prevention but also for resource allocation via targeted screening within given populations. This shift to a more dynamic, algorithmic approach will improve, if not radically alter, population screening programmes from both scientific and social perspectives.

In 1968, the WHO in its Principles and Practice of Screening for Disease set the criteria for modern screening programs in populations [3]. Around the world, the general criteria for establishing a screening programme still include the classical components of the importance of the condition, an acceptable and suitable test (clinical utility), the availability and acceptability of treatment, a policy on whom to treat, the availability of screening facilities, and continuity [4]. The WHO criteria have since been refined to also include the importance of an acceptable balance between benefit and harm, integrated monitoring and evaluation (cost-effectiveness), equity, and informed choices [5]. With the exception of newborn screening programmes that seek to find asymptomatic, at-risk newborns and that are generally considered to be in the best interests of all children, participation in population screening remains voluntary.

Screening finds apparently well persons in a given population who may have a disease and who are then individually tested. Screening can also identify persons with an increased susceptibility to a genetic disease, but screening is not a diagnostic test. Screening is applied across populations for diseases for which early detection and treatment can prevent or at least, ameliorate the consequences. Screening can also be applied to sub-populations, such as women over 50 years of age who are routinely offered breast cancer screening. Pap smear and mammography screening initiatives date back to the 1950–1960s. 

The intention of any cancer screening programme is not merely to detect cancers but also to treat them so as to reduce deaths. Not all individuals involved in screening benefit, and in fact, some can be harmed. The trade-offs include missing cancer diagnosis or subjecting an individual to unnecessary investigation, overdiagnosis, and overtreatment. To date, several studies in breast and prostate cancer have reported that tailoring screening to an individual’s risk level could improve the efficiency of the screening program and reduce its adverse consequences [6,7,8].

At present, the mammographic breast screening programmes for the general population have age as the only entry criterion. The starting and stopping ages (varying from 40 to 74 years) and the frequency of screens (yearly to triennially) differ between countries [9].

The risk of developing breast cancer varies among women. There are different subtypes of breast cancer, and the growth rate of breast cancers, even of the same subtype, varies widely, from being almost static to fast-growing [10]. The age-based or the “one-size-fits-all” approach, however, does not take into account the heterogeneity of the breast cancer subtypes, biological behaviour, and the risk in the population.

Fast growing cancers quickly lead to symptoms and death. Uniform screening would miss detecting fast growing tumours. Some tumours grow at a slow enough pace that the individual would die from other causes before the cancer manifests symptoms. As mentioned, detecting these tumours and treating them does not necessarily benefit the person but can harm them. Nonetheless, it is not possible to determine if a cancer is over-diagnosed. Identifying subgroups of individuals likely to have progressive tumours and targeting screening to them or tailoring the screening frequency and age according to their risk score could reduce the adverse consequences of screening.

The implementation of this approach must be carefully examined, including its socio-ethical and legal implications. Indeed, some would argue that the notion that risk scores will offer equivalent utility population-wide by providing informative risk stratification across multiple diseases is misleading, “raising unrealistic expectations and implementing programmes without careful evaluation risks compromising the application of risk scores for specific niches, and indeed, of genomic medicine as a whole” [11].

Another challenge inherent in implementing population health screening programmes is establishing the subsequent benefit–harm balance thereof [12,13,14]. Evidence of an appropriate benefit–harm balance can be difficult to adduce for screening programmes, as the earlier detection of disease can increase post-detection survival without decreasing cancer-specific mortality [15,16,17]. Randomised controlled trials are considered to be the gold standard for demonstrating the effectiveness of screening programmes. However, performing randomised controlled trials (RCTs) of population screening programmes is an onerous undertaking, as such trials are costly to implement and must necessarily be performed relative to a stable cohort across many years [14]. Evidence of the benefit–harm balance of a screening programme could potentially be adduced in reliance on data derived from the long-term evaluation of its functioning upon implementation, to alleviate the difficulties in performing RCTs [14].

Furthermore, the practical implementation of population screening creates further potential obstacles to establishing that the benefits thereof outweigh the harms, as the improper implementation thereof can negate the anticipated benefits [18]. Economic factors can also be relevant to the implementation of a population screening programme. The use of health-sector resources to implement and maintain a population screening programme must be justified relative to other potential uses of available resources (e.g., administrative resources, funding, labor, use of technological infrastructure) [7,14].

Assessing the benefit–harm balance of a population screening programme also requires reaching consensus on a number of social policy issues. Such policy issues include determining the most desirable balance between sensitivity and specificity (i.e., the balance of false positives to false negatives) and establishing appropriate metrics for assessing cost-effectiveness (e.g., increased screening could prevent more deaths but also could increase false findings, overdiagnosis, and use of resources). For example, a screening test can lead to improved prognosis or could lead to an improved quality of life or a less invasive course of treatment because of the earlier detection of the concerned health condition. Conversely, a false positive screening test could lead to unnecessary clinical interventions and to stress and anguish for affected persons [19]. It is also critical to address issues of equitable access and equitable outcomes in programme implementation. 

Further, the active surveillance of identified low-risk cancers is increasingly used as an alternative to surgical intervention in certain cancer treatment contexts, suggesting that accurate risk stratification is of growing relevance to clinical decision-making (e.g., for prostate cancers or thyroid cancers) [20,21,22,23,24]. Both Canada and England have successfully implemented risk-stratified approaches to the follow-up care of cancer survivors (i.e., determining the magnitude of follow-up care and whether oncologists or primary care physicians perform such follow-up) [25]. In sum, there is evidence that risk stratification could improve both the cost–benefit and risk–benefit profiles of cancer interventions that today present ambiguous cost–benefit and risk–benefit propositions, including cancer screening [26,27,28]. Presently, individual characteristics, such as age and family histories are used to determine whether screening or preventive mastectomy are liable to produce better or worse outcomes for patients [29].

In this review, we examine the socio-ethical and legal implications of risk-based breast cancer screening programmes. In particular, we study issues arising from stratification approaches that use multivariate algorithms to improve personalized risk level assessment. Such algorithmic breast cancer risk prediction models incorporate individual data, administrative health data, and possibly genetic data. Under this promising approach, women receive an estimate of a risk level or category in the form of a risk score (e.g., average, higher-than-average, high risk) (Part I). We also examine the impact of current regulatory classification of polygenic risk scores on such stratified screening programmes (Part II). 

We draw on our experience from the PERSPECTIVE I&I breast cancer project. This “Personalized Risk Assessment for the Prevention and Early Detection of Breast Cancer” seeks to improve risk-stratification to allow for cost-effective, population-based screening in Canada.

## 2. PART I: Risk Stratification: Socio-Ethical Implications

Risk stratification is a proposed method to improve the benefit–harm balance of screening programmes and other health interventions (e.g., preventive surgeries, lifestyle modification) [30,31]. The rationale is to identify high-risk individuals within a chosen population for targeted health interventions rather than to perform such interventions across the entire population. This can improve the balance of risks and benefits and the cost-effectiveness of the concerned interventions (e.g., by reducing the number of false positives and overdiagnosis of screening programmes) [32]. In ensuring that health interventions are provided to individuals that stand to benefit from them the most (i.e., through stratification), the potential negative externalities of such interventions can be minimised and the potential benefits maximised. The targeted provision of screening and other health interventions could also help to ensure that greater benefit is obtained from such initiatives relative to their costs. It must be acknowledged that certain elements of cost–benefit analysis remain inherently subjective. Competing values are engaged, including accessibility, equity, and benefit-maximization for both individuals and subpopulations [33].

Cancer care is susceptible to benefit from risk-stratified screening and other risk-stratified health interventions. Certain cancers exhibit much worse prognosis for high-risk individuals than for low-risk individuals, which implies that accurate risk stratification for the purposes of targeted intervention may be more apt to lead to improved clinical outcomes than it is to increase cancer detection [34]. Screening methodologies that are effective in cancer care are often associated with high costs or limited availability (e.g., genetic testing for BRCA 1 and BRCA 2) [35]. Preventive surgeries often impose significant burdens on patients (e.g., inherent risks or associated adverse effects) [36,37]. Information such as age, gender, family history of cancer, select biomarkers, and membership in select populations that exhibit heightened risk (e.g., persons of Ashkenazi Jewish ancestry) [38] are already relied on to personalise cancer care to anticipated risk in such subpopulations, partly in recognition of these imperatives [39,40].

In developing policies and assessment methodologies for the provision of risk-stratified healthcare, it is necessary to define the concerned population and to categorise population members according to their individuated health risk in accordance with a defined risk-stratification methodology. Methodologies for assessing individual health risk include algorithmic methodologies that entail the calculation of a risk score based on input data and human-initiated methodologies that are reliant on clinical judgment to assess health risk [41]. In practice, the most common are hybrid approaches, which involve the application of human interpretation to algorithm-derived scores [42]. Consequently, the ethical, legal, and policy issues considered relate both to the development of algorithmic risk stratification methodologies and to the application thereof by clinicians (i.e., clinical implementation).

Other related issues include ensuring that risk-stratification achieves comparable performance across sub-populations and across human genetic diversity, ensuring that individuals in different healthcare contexts obtain equitable access to risk-stratified care, and ensuring that individuals and healthcare practitioners understand their respective responsibilities in obtaining appropriate follow-up care after their risk level has been assessed.

### 2.1. Access to Data Required to Develop and Understand Risk-Stratification Algorithms 

One challenge to the creation of accurate risk-stratification algorithms relates to data access. Creating accurate risk stratification algorithms for cancer can require the combination of data from multiple sources, including clinical data, environmental data, lifestyle and behavioral data, and genomic data [43]. Contemporary initiatives have created breast cancer risk prediction models that incorporate both the presence of known pathogenic variants (e.g., BRCA 1 and BRCA 2) and polygenic risk scores that calculate a risk-score expressing the cumulative risk arising from the presence of multiple genetic variants that individually create small increases in genetic risk [44,45,46].

Large quantities of data from multiple sources are required to create accurate predictions of health risk that account for multiple potential sources of health risk (e.g., genomic data, clinical data, research data and data from biobanks, administrative health data, and aggregate population-level information) [47,48,49]. Indeed, recent research suggests that the creation of clinical risk scores for breast cancer that combine polygenic risk scores to other established biomarkers could enhance the performance of risk prediction models [28,50]. Furthermore, continuing to refine and to improve upon risk-stratified healthcare delivery will require access to rich data about the patients that participate in such initiatives and the clinical outcomes of each, for broad quality assurance and research purposes. 

Barriers to such data-sharing include non-harmonised or unclear data protection laws and data localisation requirements, which can preclude the creation of large representative datasets. Legal doctrines including collection limitation and data minimisation, purpose limitation [51,52], and strict interpretations of consent requirements and anonymisation requirements [53,54,55]—all common to data protection law—can impede the collection of rich datasets and the efficient sharing thereof. Default preclusions on inter-institutional, inter-sectoral, and international data flows can inhibit the creation of the data needed to create effective risk-stratification algorithms, as it is difficult or impossible for one institution to generate all the data needed to create such algorithms [56].

There is no doubt that tailoring screening to scores via risk stratification not only will foster more targeted care but also will help to promote the economic sustainability of universal health care systems. To that end, age-based programmes require re-examination as does screening frequency, to say nothing of accounting for human genetic diversity. 

### 2.2. Risk Stratification across Human Genetic Diversity

Another challenge for the clinical implementation of stratified health risk is the need to produce estimates that can be generalised for individuals exhibiting different genetic ancestry or coming from different population groups. Risk stratification increasingly leverages genetic data. Human genetic composition and determinants of genetic risk or hereditary risk differ considerably across different human ancestry groups [57].

Health disparities in access to personalised risk stratification levels could arise across different ancestral groups due to a lack of access to rich genomic data concerning such ancestry groups that are often underrepresented in population health databases or large-scale genetic databases (i.e., data concerning individuals that do not exhibit European genetic ancestry) [2,58]. If insufficient input data are available for individuals of an ancestry group, it is probable that a risk stratification algorithm would be less effective for that group than for the larger population. Genetic ancestry groups that are underrepresented in available data could therefore be subject to less accurate risk stratification than are genetic ancestry groups that are traditionally well-represented in biomedical datasets.

To ensure that risk stratification algorithms can produce accurate risk-estimates for all members of society, a number of approaches might be practicable. Possibilities discussed in the scientific literature include the creation of separate PRS algorithms or risk stratification methodologies for individuals from distinct genetic ancestry groups, as well as the creation of a singular algorithm trained on holistic training data that are representative of diversity in genetic ancestry (i.e., PRS scores with cross-population portability) [59,60,61,62,63,64,65,66,67]. In either instance, it will be necessary for large quantities of rich data from diverse human populations to be made available to health sector entities to ensure that risk stratification methodologies yield equitable and applicable results [68].

### 2.3. Equitable Access to Risk Stratification 

Ensuring equitable access to risk stratification programmes is another considerable policy challenge. This is difficult because specialised staff, technological infrastructures, and considerable data storage are required to implement and maintain risk-stratification programmes. Such infrastructures may be accessible in the major metropolitan health centres but could be inaccessible in healthcare centres located in rural areas or in healthcare institutions situated in developing economies [69,70]. There is no doubt that infrastructural investment in the informatics resources and personnel needed to support personalised medicine should be adopted in tandem with risk stratification programmes.

Effective communication between members of the healthcare team is also required to successfully act upon a heightened health risk [71,72]. If healthcare practitioners cannot effectively ensure that the identification of heightened health risk leads to beneficial changes in healthcare pathways or in patient decision-making, the enterprise of risk-stratified care provision could be threatened [73,74]. It is therefore critical that health institutions and health professionals understand and be able to act on the knowledge gained from risk profiling. 

### 2.4. Long-Term Follow-up for Risk-Stratified Patients 

Another policy challenge for cancer risk stratification is the need to establish expectations concerning the future care of screening participants who have been stratified into specific risk levels. It is important to communicate whether the risk profile obtained is anticipated to remain static in the future or whether changes in the individual’s health or lifestyle or in the state of medical knowledge could prompt the recalculation of personalised risk estimates. Certain risk factors are liable to change throughout the lifecycle (e.g., risk factors related to lifestyle choices and environmental exposures) [75]. Other assessed risk factors could be subject to changed interpretations in the future, such as genetic variants, the interpretation of which is susceptible to change as scientific consensus evolves (i.e., variant reclassification) [76].

Screened individuals should be informed of whether changes in their risk profile will be communicated to them absent an intervention (e.g., in the instance that medical knowledge evolves) and whether they should return for their risk category to be calculated anew. It is dubious that the legal duties of healthcare practitioners include the obligation to recontact screening participants to inform them of changes in their risk level resulting from changes in scientific knowledge (e.g., a genetic variant reclassification or a modified personal risk score arising from a change in the method used to assess genetic risk) [77,78]. A potential duty to update individuated risk profiles could however arise if a healthcare practitioner learns about novel information that clearly changes prognosis or the treatment path for a specific patient under the healthcare practitioner’s care [77,78]. 

Nonetheless, healthcare practitioners should clearly establish respective responsibilities for ensuring the re-calculation and updating of risk levels in collaboration with patients [31]. Empirical research drawn from the literature of genetic recontact suggests that patients in the United Kingdom could anticipate that their physician will contact them to update them as to changes in their health risk, unless told otherwise [79]. However, such expectations might not be held in other countries in which most individuals do not have an active and ongoing relationship with a primary care physician or with specialised genetic care providers.

It also bears noting that patients have an established ethical right to decide whether or not to be informed of new information that is relevant to their health status (i.e., the right to know and its corollary, the right not to know). Therefore, it is prudent to dialogue with patients to establish respective obligations to update individual health risk [80]. In the future, it is possible that physicians could have a legal duty to alert patients to significant changes in their health risk if new technologies were to enable the seamless integration of such communication to clinical workflows (e.g., the advent of learning health systems enabling the automated dissemination of updated risks scores to patients) [81].

## 3. PART II: Polygenic Risk Scores: Regulatory Implications

Genome-wide association studies (GWAS) have uncovered the relevance of inherited variants to common complex diseases, furthering the integration of genetic data within risk score algorithms [82,83]. Most non-communicable disorders have a genetic component that comprises hundreds or thousands of genetic variants, each of which has a small effect on the disease risk [83]. While genetic testing is widely used to diagnose monogenic diseases determined by mutations in a single gene, polygenic disorders are caused by many genetic variants located throughout the whole genome, as well as by environmental and lifestyle factors [84]. Each of these variables is valuable in the pathway of the disorder, but it is not informative for assessing the overall disease risk [82,83]. A PRS is a weighted sum of several of the risk variants for a particular disease [83,84]. It provides an estimate of an individual’s genetic vulnerability to a trait or disease [82]. In other words, PRSs are the tool by which the knowledge of these common variants can be used to improve healthcare by providing a point of reference that could place an individual is a lower-than-average, average, or above-average risk category and therefore potentially improve screening, advance preventive medicine, and achieve a more personalized treatment [12,83]. The estimate provided by PRSs is calculated based on the individual’s genotype profile in comparison with the relevant GWAS data [82]. The ever-growing use and availability of large quantity of genomic and health-related data from which the foregoing can be discovered and furthered, the identified advantages of preventive medicine, and the increasing personalization of medicine have emphasized the use and potential usefulness of polygenic risk scores (PRS) in risk stratification and screening practices and programmes [85].

Considering the role that PRSs play and continue to play in programmes and practices of screening and stratification involving genomic data, any of the implementation, adoption, and development issues related to PRSs are therefore of paramount importance while discussing those of programmes and practices of screening and stratification involving genomic data. The regulatory framework applicable to PRS as a non-device clinical decision support tool or as a medical device is one of the most crucial systemic implementation issues. Regulatory frameworks differ in this context.

### 3.1. Regulatory Framework

Medical device (MD) regulatory frameworks ensure that MDs brought to market are safe and effective. They do so by evaluating their quality, effectiveness, accuracy, and safety [86,87]. PRSs keep evolving, moving from research discovery studies to clinical research studies and to possible clinical implementation [83]. However, their clinical utility and analytical validity are still to be improved and refined for wider clinical use in risk prediction, treatment response, and prognosis [83]. These scientific and technical limitations complicate their current definition within the regulatory frameworks, as their use for medical purposes is uncertain under the law [12,85]. This uncertainty extends to all jurisdictions. Furthermore, this uncertainty is exacerbated because, despite increasing efforts, MD regulatory frameworks are not internationally harmonised. The regulatory processes (requirements, costs, timelines, risk classes) as well as their applicability to the specific device vary across jurisdictions. While in some jurisdictions, PRSs could be deemed to fall under MD regulatory oversight, in others, they would not [88,89,90,91,92]. Moreover, as MDs, they could be considered standalone MDs or accessories, or even in vitro (IV) MDs [85,93].

If the PRS were to fall within the oversight of MD regulation, it would impose timelines, costs, and stringent and formal requirements concerning the clinical evaluation and safety and performance certifications on the manufacturer for the PRS to be placed in the market [92,94,95,96,97,98]. The stringent and formal requirements involve hiring specialized services to prepare the supporting documentation to attach to the application file. The costs associated with obtaining MD licenses/certifications include administrative fees for the regulatory agency to receive, evaluate, and issue the license/certification; the preparation of supporting documentation; and annual renewals (in some jurisdictions). These costs vary depending on the risk class assigned (out of four different risk classes), the type of MD (MD or IVMD), and the jurisdiction (we reviewed Canada, the US, and the EU): they can range between USD 300 and 12,000 for the administration fees, USD 300 for annual fees, and USD 3000 and 8000 for the preparation of supporting documentation [99,100,101,102,103]. The timelines to undergo the regulatory approval process can range from 15 and 177 days, depending on the same parameters as with respect to the costs [104,105,106,107,108,109].

Where PRSs are not regulated as MDs, PRSs would be considered non-device clinical decision support tools. Clinical decision support refers broadly to tools that “provide healthcare professionals and patients with knowledge and person-specific information, intelligently filtered or presented at appropriate times, to enhance health and healthcare” [90]. Tools that support diagnosis are one example. In this case, MD regulations do not apply. Manufacturers and researchers are instead encouraged to follow best practices for validation and quality assurance (e.g., ISO standards related to health software, medical device software, medical devices). 

Subjecting PRSs to a MD regulatory framework would have enormous implications on the use, implementation, and further development of PRSs and therefore on the associated stratification and screening programmes. We identified two main implications for such a classification: the further development of PRS and access thereto. 

### 3.2. Further Development of PRS

Many aspects of the complexity, interpretation, understanding, and significance of the information provided by PRSs still need to be refined, validated, and translated into clinical tools and models via ongoing research [12]. For instance, despite the growing association between PRS and disease status in research-based case-control studies, the clinical utility of PRSs is yet to be established [83]. For PRSs to be used by clinicians or researchers for individual diagnosis, prediction, and stratification, relative risks will need to be transformed to absolute risks [83]. The focus of PRSs would then have to be able to provide information for a single individual [83]. In complex disorders, there are multiple factors that contribute to the disease, all of which need to be properly understood in order to avoid imperfect or incorrect measures and reactions that could give false impressions of genetic determinism and could harm health decisions. Likewise, while currently unknown, PRSs could have a role in treatment response and in refining the penetrance of high-risk variants [83]. Moreover, as we have already mentioned, health disparities may be exacerbated by the focus of GWAS studies on individuals of European ancestries and the under-representation of the ancestries of minority ethnic groups and low- and middle-income countries. Current PRSs have limited clinical applicability in minority ethnic groups and low- and middle-income countries, as their specific ancestries are usually under-represented in the databases used to calculate their risk scores [83]. Improving the scientific validity, clinical utility, and clinical validity of PRSs, as well as increasing the ancestry diversity, greatly depends on global collaboration. 

Uncertain or burdensome regulatory frameworks may discourage collaboration in some cases, as potential collaborators or funders may not want to have to face any potential consequence of that uncertainty or burden. Likewise, if PRSs were to be deemed subject to the MD regulatory framework, the resources that their creator(s) and/or funders need to invest, as well as the efforts and time that they would need to devote, could very likely disrupt the current practices of open and broad sharing associated with PRSs and prompt instead a proprietary attachment to the PRS methodology and knowledge. For instance, this kind of proprietary attachment and perspective could lead to pursuing forms of intellectual property, which would encourage and enable restrictive, complicated, and burdensome terms to use, share, and/or collaborate with PRSs. Some of these terms could involve charging licensing fees or royalties to potential collaborators or users, which could unnecessarily complicate, extend, and raise the costs of collaborations. Uncertain, complicated, and/or costly collaboration caused either by MD regulatory frameworks or forms of intellectual property could reduce the number of potential collaborators. A reduced network of collaborators could disrupt or decelerate the development of PRSs, their clinical utility, and their overall translation into the clinical setting [85]. Reduced collaboration could also limit the peer-reviewed accuracy and improvement of the different methods used to calculate the PRSs. Likewise, complicated and costly collaboration could also delay the inclusion of diverse ethnic groups and the applicability of PRSs to non-European populations.

Furthermore, the risk arising from an individual’s genetic information is dynamic, as it depends on and changes with other factors such as age, environmental exposures, and the exposure to other illnesses [12,83]. Consequently, despite the risk stratification provided by an individual’s genetic information, if that individual is not exposed to the other factors, the relevance of the genetic risk may change [83]. This dynamism requires a framework that is sufficiently flexible for the risk scores and PRSs methodology and calculations to adapt to these changes in order to provide an accurate risk assessment without having to obtain new regulatory authorizations. The current MD regulatory frameworks do not offer this kind of flexibility, as most changes in the regulated devices bring about the need for new assessments to determine whether the risk class has changed and whether the device continues to be safe and effective. 

### 3.3. Access

Burdensome and lengthy regulatory frameworks can negatively impact access to PRSs in two ways: delaying the availability of PRSs and limiting patient access thereto. If MD regulatory frameworks were to oversee PRSs, the latter would need to meet all the applicable requirements and obtain the necessary licenses and authorizations before they could be brought to market. Until these requirements are met and licenses are obtained, populations would have to wait to be able to benefit from the use of PRSs. Burdensome and lengthy regulatory frameworks would extend this wait time [85]. This delay would be exacerbated by the fact that regulatory frameworks are not harmonised among jurisdictions. Furthermore, given the costs associated with the compliance the regulatory frameworks in each jurisdiction, these added costs could discourage the introduction of the PRS to specific markets. The proprietary approach that burdensome and costly regulatory frameworks could prompt is also likely to impact the prices to access PRSs, creating inequitable access to PRSs and therefore to healthcare. 

The detrimental effects that ill-suited, convoluted, or uncertain regulatory frameworks could have on the development, implementation, use, and access of PRSs could also extend to those of other risk assessment tools. One of these tools could be risk prediction models, which can incorporate PRSs combining clinical, biochemical, lifestyle, and historical risk factors to predict 10-year risk of cardiovascular disease, diabetes, or breast/ovarian cancer [12,40,83,85,110,111,112,113]. As such, inappropriate or uncertain regulatory frameworks could prevent patients from having access to available PRSs, risk prediction models, or screening programmes that could enable timely diagnoses that could offer the opportunity to make simple changes in their lifestyles or more moderate adjustments such as diet or exercise instead of undergoing more radical course of action such as surgical procedures or pharmacological interventions [83]. CanRisk is one of these risk prediction models.

While much of the research on PRSs comes from an interest on predicting and offering better screening and treatment for patients with cardiovascular diseases, type 2 diabetes, breast, cervical, and prostate cancer, Alzheimer’s disease, and psychiatric disorders [83,114], the strongest clinical utility has been found in cardiovascular diseases and in breast cancer. Even in these cases, there are still issues with respect to the applicability of these risk assessments across ethnic groups. This means that there are still several scientific, technical, and implementation issues that need to be resolved. Regulatory frameworks must provide certainty about the regulatory path and strike a balance between ensuring safety and encouraging wide collaboration and equitable use. 

Finally, the legal classifications used by the regulators, that is, medical devices versus non-medical device clinical support tools, are not “value neutral”: The socio-economic, equity, and professional implications of classification algorithms as medical devices versus the professional standards approach of clinical support tools has global implications for healthcare system costs, access, and use. It is self-evident that screening for common diseases and ensuring the appropriate stratification of such efforts remain health goals of public importance. Therefore, risk-stratified care performed in reliance on PRS scores requires further policy consideration. The same can be said of the ensuing professional obligations related to risk-stratification tools, and the possible commercialisation of stratification tools such as PRSs. Yet, there is no doubt that screening, stratification, and risk scores have yet to enter common, professional, public, and political parlance. 

## 4. Conclusions

In closing, it bears mention that the clinical implementation of personalised medicine in health care systems, including risk-stratified medicine, will require the secondary use of coded health data to be performed as a default proposition rather than as an exception to the general non-use of health data [115,116]. Legislators and regulators need to provide clarification as to the potential to collect, retain, use, and share rich datasets for the purpose of improving screening and healthcare delivery. Not all data protection laws make clear the lawfulness of doing so; ambiguities regarding the potential to keep and to share such data can inhibit the development of tools for personalised medicine [117].

Institutions participating in the creation of risk-stratification algorithms or input datasets should create practices and procedures for the generation and sharing of data in collaboration with other institutions that are host to relevant datasets. The adoption of shared policies and the creation of collaborative data commons can enable the creation of large datasets to serve as training data for risk stratification algorithms, even if legal standards are not harmonised in the jurisdictions of all collaborators [118]. Likewise, it is important to create and promote clear and harmonised regulatory frameworks that enable both the scientific advancement and safety of risk assessment and stratification tools, such as the PRS and risk prediction models that facilitate those stratification and screening programmes.

In the long term, the adoption of concerted national and international institutions dedicated to the clinical implementation of personalised medicine and to the creation of common platforms for data storage, harmonisation and interpretation are necessary [119]. Such bodies could support local institutions in integrating risk-stratified care to their local practice and could participate in collecting and analysing the large evidence-base needed to perform effective population screening and risk-stratification, in compliance with ethical and legal norms [120]. Such centralised support could assist in the development of standard practices to ensure that data are collected and interpreted in interoperable formats [121]. This support could also help delegate responsibilities for data interpretation to appropriate specialists, thereby reducing the burden of data collection and interpretation by health professionals and ensuring effective screening, stratification, and communication of risk scores throughout the health system [122].

## Data Availability

Data sharing not applicable; No new data were created or analyzed in this study. Data sharing is not applicable to this article.
